# Loss of CRY2 promotes regenerative myogenesis by enhancing PAX7 expression and satellite cell proliferation

**DOI:** 10.1002/mco2.202

**Published:** 2023-01-09

**Authors:** Yingxue Hao, Ting Xue, Song‐Bai Liu, Sha Geng, Xinghong Shi, Panting Qian, Wei He, Jiqing Zheng, Yanfang Li, Jing Lou, Tianze Shi, Ge Wang, Xiaoxiao Wang, Yanli Wang, Yangxin Li, Yao‐Hua Song

**Affiliations:** ^1^ Cyrus Tang Hematology Center Collaborative Innovation Center of Hematology Soochow University Suzhou P. R. China; ^2^ National Clinical Research Center for Hematologic Diseases The First Affiliated Hospital of Soochow University Suzhou P. R. China; ^3^ State Key Laboratory of Radiation Medicine and Protection Soochow University Suzhou P. R. China; ^4^ Suzhou Vocational Health College, Suzhou Key Laboratory of Biotechnology for Laboratory Medicine Suzhou Jiangsu P. R. China; ^5^ Institute for Cardiovascular Science and Department of Cardiovascular Surgery First Affiliated Hospital and Medical College of Soochow University Suzhou Jiangsu P. R. China; ^6^ Collaborative Innovation Center of Hematology Soochow University Suzhou Jiangsu P. R. China

**Keywords:** CRY2, muscle regeneration, satellite cells

## Abstract

The regenerative capacity of skeletal muscle is dependent on satellite cells. The circadian clock regulates the maintenance and function of satellite cells. Cryptochrome 2 (CRY2) is a critical component of the circadian clock, and its role in skeletal muscle regeneration remains controversial. Using the skeletal muscle lineage and satellite cell‐specific CRY2 knockout mice (CRY2^scko^), we show that the deletion of CRY2 enhances muscle regeneration. Single myofiber analysis revealed that deletion of CRY2 stimulates the proliferation of myoblasts. The differentiation potential of myoblasts was enhanced by the loss of CRY2 evidenced by increased expression of myosin heavy chain (MyHC) and myotube formation in *CRY2^−/−^* cells versus *CRY2^+/+^* cells. Immunostaining revealed that the number of mononucleated paired box protein 7 (PAX7^+^) cells associated with myotubes formed by *CRY2^−/−^* cells was increased compared with *CRY2^+/+^* cells, suggesting that more reserve cells were produced in the absence of CRY2. Loss of CRY2 leads to the activation of the ERK1/2 signaling pathway and ETS1, which binds to the promoter of PAX7 to induce its transcription. CRY2 deficient myoblasts survived better in ischemic muscle. Therefore, CRY2 is essential in regulating skeletal muscle repair.

## Graphical abstract

Schematic diagram of signaling pathways mediating PAX7 expression induced by CRY2 deletion. CRY2 deletion led to the activation of JNK1/2 and ERK1/2 signaling pathways, activating ETS1 to promote the transcription of PAX7 and the activation of satellite cells.

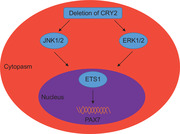

## 1 INTRODUCTION

Regenerative myogenesis relies on the proliferation and differentiation of satellite cells. Under physiological conditions, satellite cells are quiescent, but they are activated upon muscle injury and become proliferating myoblasts characterized by the expression of MyoD.^1^ The satellite stem cell pool is maintained by self‐renewal, which was documented by single myofiber transplantation experiment showing that a few satellite cells can generate numerous new myofibers.^2^ Both symmetric and asymmetric division contribute to self‐renewal, depending on the surrounding microenvironment. The symmetric division produces two identical satellite cells, whereas the asymmetric division produces one satellite cell and one destined for differentiation.^3^

The paired box protein 7 (PAX7) is critical for the survival of satellite cell.^4^ The loss of PAX7‐expressing cells blocks myogenic regeneration.^5^, ^6^

MyoD is one of the transcription factors in directing satellite cells toward myoblast lineage.^7^ Activated satellite cells proliferate and form clusters that express PAX7 and MyoD (PAX7^+^/MyoD^+^), which then become PAX7^−^/MyoD^+^ cells as PAX7 is downregulated.^8^ In contrast, a small percentage of the PAX7^+^/MyoD^+^ satellite cells return to quiescence by downregulating MyoD instead of PAX7.^8^ Overexpression of PAX7 inhibits the expression of MyoD and myogenin. However, the mechanisms underlying satellite cell fate decisions remain largely unknown.

Emerging evidence suggests that satellite cell activation and differentiation are affected by the circadian clock, which include BMAL1, CLOCK, CRY, and PER. The expression of CRY and PER is positively regulated by BMAL1 and CLOCK. However, CRY and PER negatively regulate the expression of CLOCK and BMAL1 by inhibiting their transcription. Genomic deletion of BMAL1, CLOCK, or the Per genes results in reduced muscle mass and locomotor activity,^9^, ^10^, ^11^, ^12^ whereas the deletion of CRY1/CRY2 enhances exercise capacity.^13^ However, a subsequent study showed that CRY1 and CRY2 have differential roles in regulating muscle regeneration.^14^ These conflicting reports regarding the role of CRY2 on skeletal muscle function may be explained by the fact that these studies used germline CRY knockout mice. The phenotype of these mice could be the results of developmental changes in multiples tissues. To address this issue, we generated skeletal muscle lineage and satellite cell‐specific CRY2 knockout mice (CRY2^scko^). We show that the deletion of CRY2 enhances the regeneration of injured myofibers. Our data demonstrate that the deletion of CRY2 in satellite cells activates ERK1/2 signaling pathway, which induces the phosphorylation of the transcription factor ETS1, which in turn activates the transcription of PAX7. Single myofiber analysis showed that deletion of CRY2 enhances satellite cell self‐renewal. CRY2 deficient myoblasts had a higher survival rate than the control when transplanted into ischemic muscle. Therefore, CRY2 deletion enhances satellite cell function.

## 2 RESULTS

### 2.1 CRY2 deletion promotes myogenic regeneration

**FIGURE 1:**
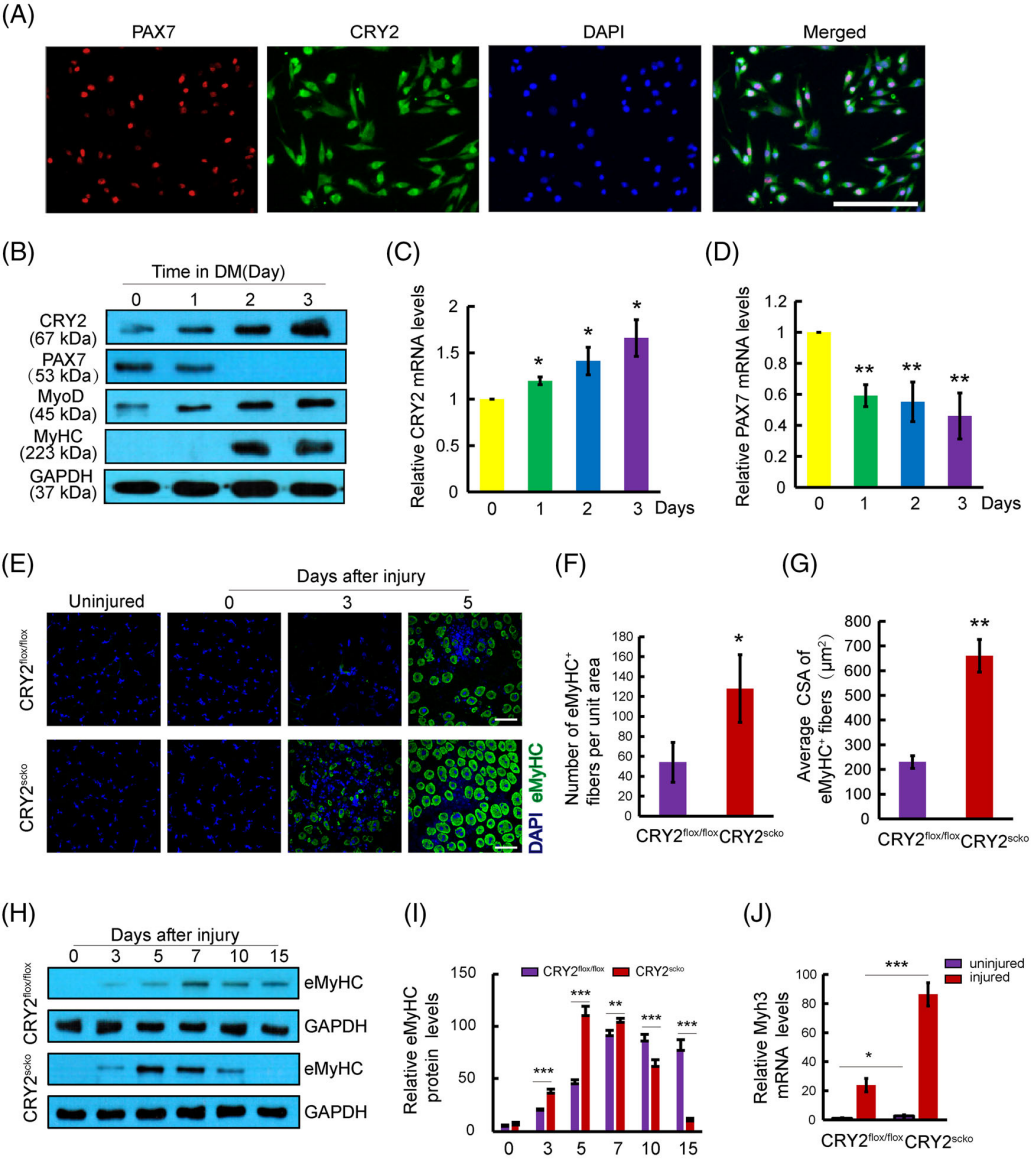
Localization of CRY2 and the impact of deletion of CRY2 on the expression of eMyHC. (A) Immunostaining of CRY2 (green) and PAX7 (red) in primary myogenic cells. Nuclei were labeled by DAPI (blue). Scale bar: 50 μm. (B) Western blot analysis of the levels of CRY2, PAX7, MyoD, and MyHC in primary myogenic cells cultured in a differentiation medium containing 2% horse serum. GAPDH was used as the loading control. (C and D) mRNA levels of CRY2 and PAX7 in primary myoblasts cultured in the differentiation medium for 3 days. *p*‐Values were determined by ordinary one‐way ANOVA with Dunnett's multiple comparison test. (E) Immunostaining of eMyHC (green) for regenerating myofibers in TA muscles from CRY2^flox/flox^ and CRY2^scko^ mice at different time points after BaCl_2_‐induced injury and uninjured muscles. The nuclei were stained with DAPI (blue). Scale bar: 50 μm. (F and G) Quantification of the number of eMyHC^+^ fibers per field (0.08 mm^2^), and average cross‐sectional area (CSA) of eMyHC+ fibers in TA muscles 5 days after injury. *p*‐Values were determined by unpaired Student's *t*‐test. (H) Western blot analysis of eMyHC in TA muscles of CRY2^flox/flox^ and CRY2^scko^ mice at different time points after BaCl_2_ injection. (I) Quantification of relative eMyHC protein levels. *p*‐Values were determined by unpaired Student's *t*‐test. (J) Real‐time quantitative PCR (RT‐qPCR) analysis of relative mRNA levels of Myh3 (eMyHC mRNA) in uninjured and injured TA muscles (3 days). *p*‐Values were determined by unpaired Student's *t*‐test. *n* = 3 mice per group. The mice that are used for these experiments were 6–8 weeks of age. *p < 0.05, **p < 0.01, ***p < 0.001. Data are presented as mean ± SD.

Primary myoblasts were immunostained using antibodies against PAX7 and CRY2. Confocal microscopy revealed that PAX7 is present in the nucleus, whereas CRY2 can be found in both the cytoplasm and nucleus (Figure 1A). The purity of the myoblasts was verified by MyoD staining, and the data show that most of the primary myoblasts are MyoD^+^ (Figure S1A,B).

Myoblasts can differentiate and form myotubes when plated at high confluency in the differentiation medium. Myotube formation is associated with reduced PAX7 expression and increased levels of MyoD, myogenin, and myosin heavy chain (MyHC).^15^ To explore the potential relationship between CRY2 and myogenic markers, the expression of CRY2, PAX7, MyoD, and MyHC during differentiation was analyzed. Western blot analysis revealed increased protein levels of CRY2, MyoD, and MyHC, whereas the level of PAX7 was reduced (Figure 1B). These results were verified at the mRNA level by real‐time quantitative PCR (RT‐qPCR) (Figure 1C,D). Furthermore, CRY2 overexpression suppressed PAX7 expression (Figure S1C,D). This indicates that CRY2 negatively regulates PAX7 expression.

**FIGURE 2:**
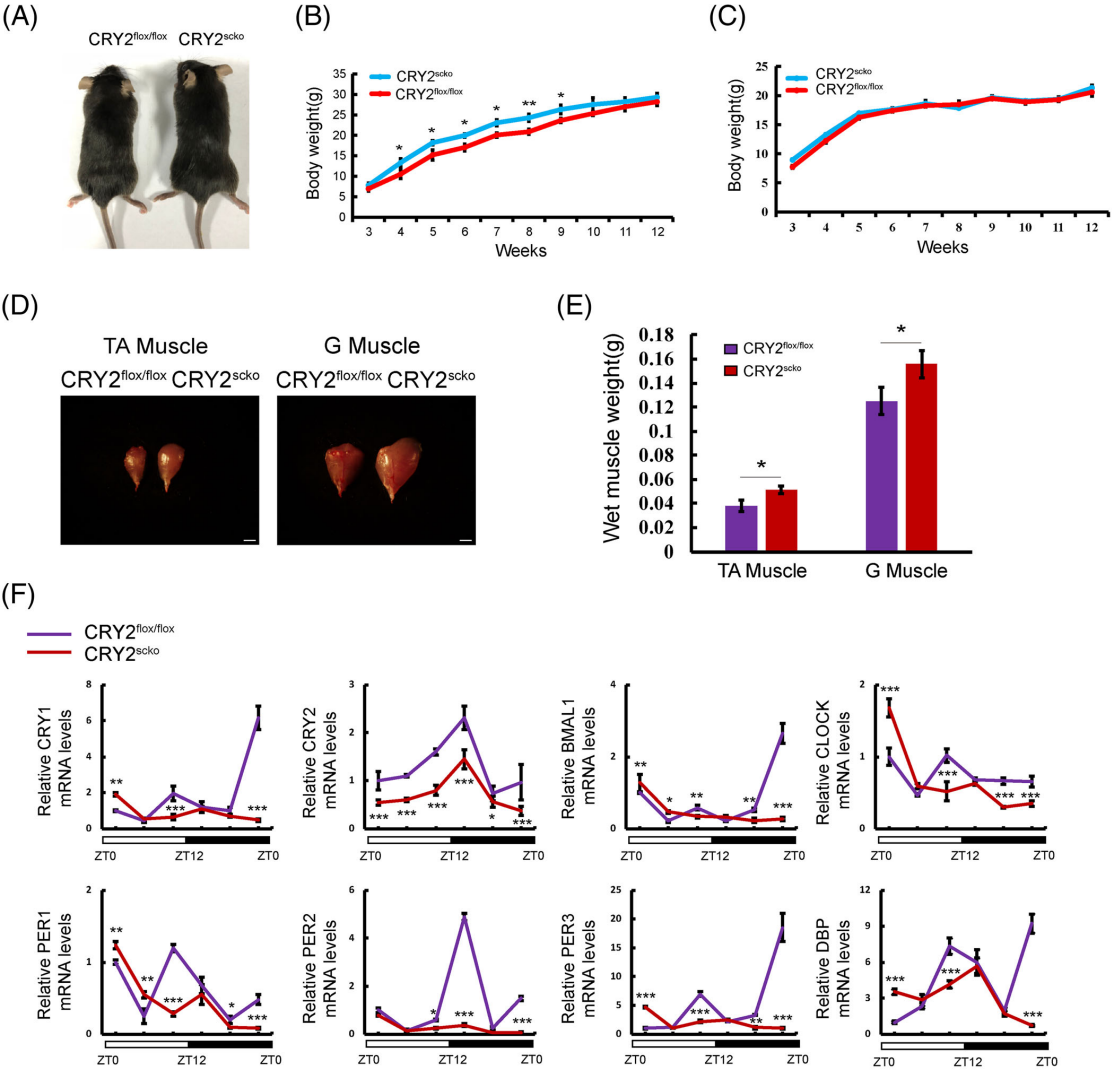
The phenotype of CRY2^scko^ mice. (A) Representative photographs of CRY2^flox/flox^ and CRY2^scko^ mice of 6–8 weeks. Growing curve of male (B) and female (C) CRY2^flox/flox^ and CRY2^scko^ mice. (D) Representative photographs of TA and gastrocnemius muscles isolated from CRY2^flox/flox^ and CRY2^scko^ mice of 6–8 weeks. Scale bar: 2 mm. (E) Wet muscle weigh of (D). *n* = 6 mice in each group at different time points for body and muscle weight. (F) Expressions of circadian clock genes in the TA muscles from CRY2^flox/flox^ and CRY2^scko^ mice of 6–8 weeks. The light was on between zeitgeber time 0 (ZT0) and ZT12. *n* = 3 independent experiments. *p*‐Values determined by unpaired Student's *t*‐test. *p < 0.05, **p < 0.01 ***p < 0.001. Data are presented as mean ± SD.

To determine whether CRY2 is involved in myogenesis, we generated skeletal muscle lineage and satellite cell‐specific CRY2 deletion (CRY2^scko^) mice by mating CRY2^flox/flox^ mice with *PAX7‐Cre* mice (Figure S2A). The identity of the CRY2^scko^ mice was verified by PCR (Figure S2B). The efficiency of CRY2 deletion in myoblasts was confirmed at both mRNA and protein levels (Figure S2C,D). The body and muscle weights of 8‐week‐old male CRY2^scko^ mice are higher than those of control mice (Figure 2A,B,D,E), whereas the body weight of the female did not differ (Figure 2C). We measured the expression of the clock genes within a 24‐h period by qRT‐PCR. The data revealed a significant downregulation of gene expression levels of both CRY2 and Per2, with delayed rhythm peaks in other core circadian clock genes in *CRY2^−/−^* versus *CRY2^+/+^* cells (Figure 2F).

**FIGURE 3:**
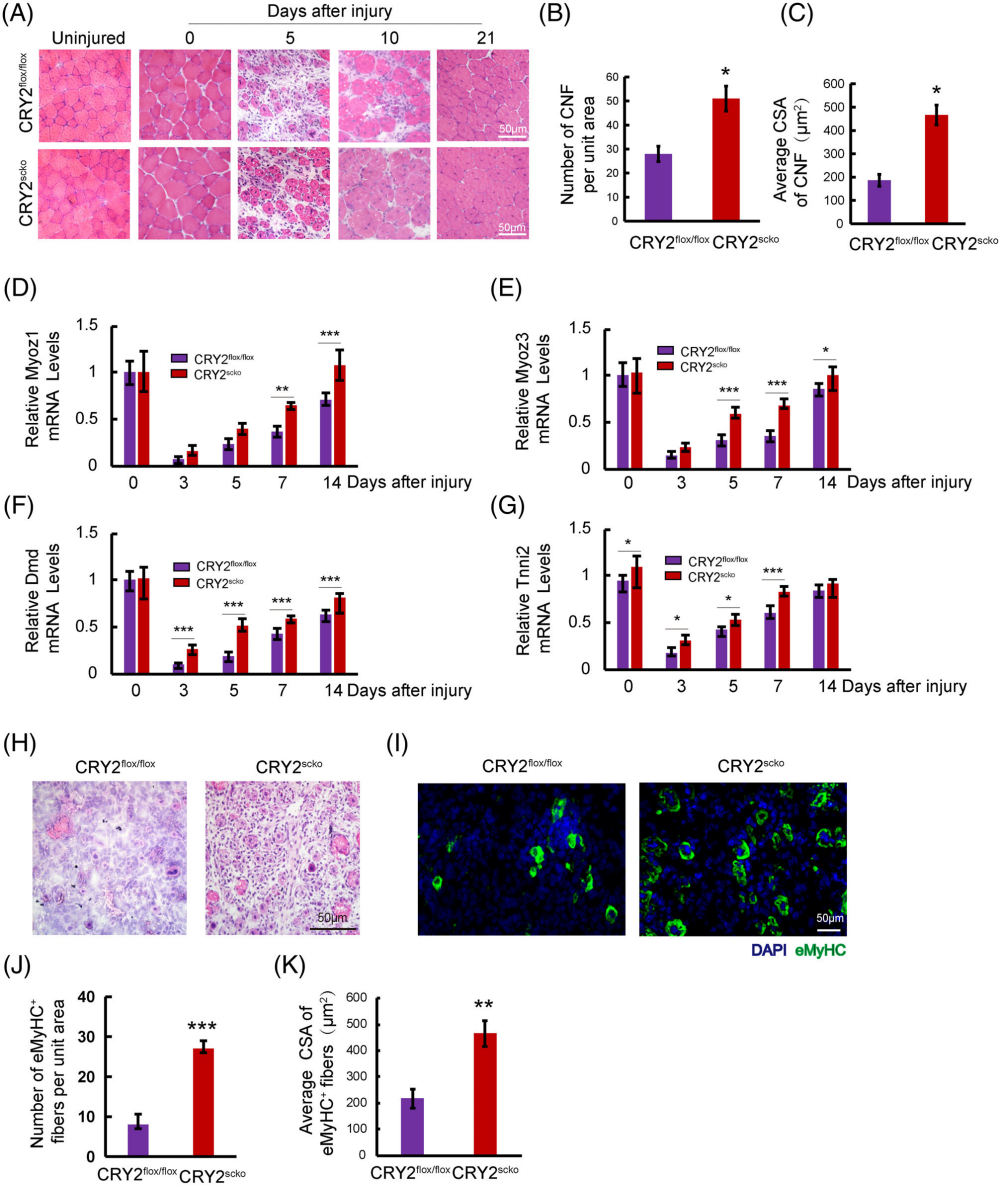
Loss of CRY2 enhances skeletal muscle regeneration. (A) Hematoxylin and eosin (H&E) staining of TA muscles from CRY2^scko^ and CRY2^flox/fox^ littermates after BaCl_2_ injection and uninjured muscles. Scale bars: 50 μm. (B) Numbers of centronucleated fibers (CNF) per field (0.04 mm^2^) on day 5 post‐injury. (C) Average cross‐sectional area (CSA) of CNF. Real‐time quantitative PCR (RT‐qPCR) analysis of mRNA levels of Myoz1 (D), Myoz3 (E), Dmd (F), and Tnni2 (G). (H) H&E staining was performed on frozen sections of TA muscles after the second injury. TA muscles of CRY2^flox/flox^ and CRY2^scko^ mice were injected with BaCl_2_ again 21 days after the initial injury, and the muscles were isolated at day 5 post‐injury. Scale bars: 50 μm. (I) Immunofluorescence staining of eMyHC (green) of TA muscles after the second injury. Nuclei were stained with DAPI (blue). Scale bars: 50 μm. (J) Number of eMyHC^+^ fibers per unit area (0.04 mm^2^). (K) The average CSA of eMyHC+ fibers. *n* = 3 mice per group. Mice used to start the experiment were pooled at 6–8 weeks of age. *p*‐Values determined by unpaired Student's *t*‐test. *p < 0.05, ** *p* < 0.01, *** *p* < 0.001. Data are presented as mean ± SD.

To determine how CRY2 deletion affects muscle repair, we injected BaCl_2_ solution to induce acute injury in TA muscle. Hematoxylin and eosin (H&E) staining revealed that the number of centronucleated fibers (CNF) and cross‐sectional area (CSA) of CNF was increased in the TA muscle of CRY2^scko^ mice as compared with that of littermate CRY2^flox/flox^ mice (Figure 3A–C). We then examined the mRNA levels of several genes related to muscle regeneration, including Myoz1, Myoz3, Dmd, and Tnni2 in uninjured and injured TA muscles^16^ and found that their expression is higher in the CRY2^scko^ mice versus the CRY2^flox/flox^ mice, suggesting an accelerated regeneration resulting from the loss of CRY2 (Figure 3D–G). To determine how CRY2 deletion affects muscle regeneration in repetitive injury, BaCl_2_ was injected again into the same TA muscle 21 days after the first injury. H&E staining was performed again 5 days after the second injury, and the results revealed an increased number of CNF in the CRY2^scko^ mice compared with the CRY2^flox/flox^ mice (Figure 3H). To verify these results, we performed eMyHC staining, and the data revealed an increased number and CSA of eMyHC^+^ myofibers from CRY2^scko^ mice versus CRY2^flox/flox^ mice (Figure 1E–G). The expression of eMyHC peaked at day 7 post‐injury in the CRY2^flox/flox^ mice, but the peak was reached earlier at day 5 in the CRY2^scko^ mice (Figure 1H,I). The increased expression of eMyHC mRNA (Myh3) was confirmed by RT‐qPCR (Figure 1J). Therefore, the increased number of CNF and the expression of muscle regeneration‐related genes (Figure 3A–G) led to the formation of new myofibers (Figure 1E–J). The increased expression of eMyHC in CRY2^scko^ mice is also evident after the second injury (Figure 3I–K). This indicates that the regenerative ability of skeletal muscle in response to secondary injury was enhanced in the absence of CRY2.

### 2.2 CRY2 regulates the proliferation of satellite cells

**FIGURE 4:**
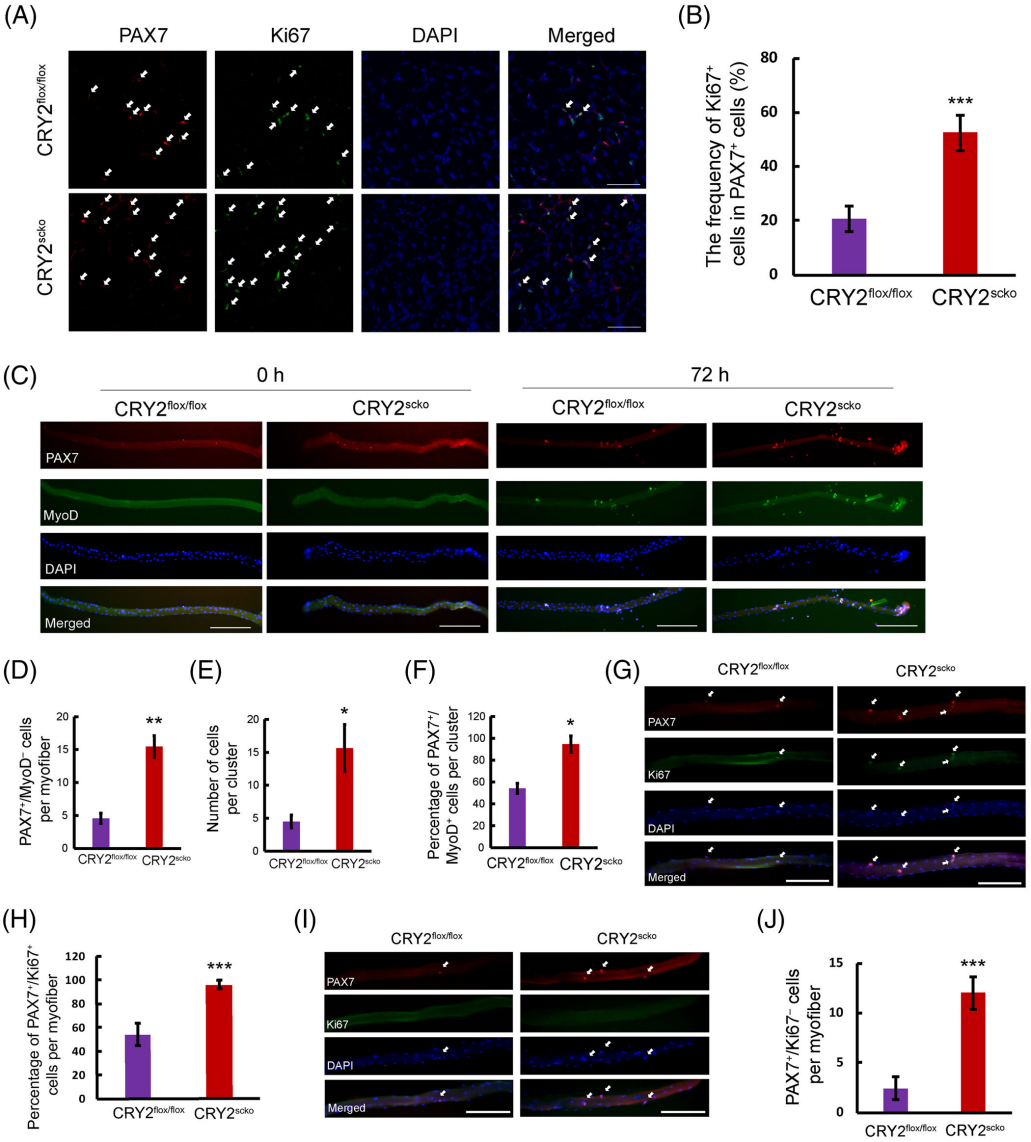
Satellite cell proliferation is enhanced in the absence of CRY2. (A) Immunostaining with PAX7 (red) and Ki67 (green) (white arrows) in TA muscles at day 5 after injury of CRY2^flox/flox^ and CRY2^scko^ mice at 6–8 weeks. Scale bar: 50 μm. (B) The frequency of Ki67^+^ cells in PAX7^+^ cells. (C) Freshly isolated (0 h) or cultured (72 h) myofibers isolated from the extensor digitorum longus (EDL) muscle of CRY2^flox/flox^ and CRY2^scko^ mice at 6–8 weeks were immunostained for PAX7 (red) and MyoD (green). Scale bar: 100 μm. (D) Quantification of the number of PAX7^+^/MyoD^−^ cells in each myofiber immediately after isolation. (E) Quantification of the number of cells in each cluster on cultured myofibers. (F) Percentage of PAX7^+^/MyoD^+^ cells per cluster. (G) Single myofibers were immunostained with PAX7 (red) and Ki67 (green) of CRY2^flox/flox^ and CRY2^scko^ mice at 6–8 weeks. Scale bar: 100 μm. (H) Percentage of PAX7^+^/Ki67^+^ cells in each myofiber. (I) Single myofibers were immunostained for PAX7 (red) and Ki67 (green) of CRY2^flox/flox^ and CRY2^scko^ mice at 8‐month‐old. Scale bar: 100 μm. (J) Quantification of the number of PAX7^+^ cells in each myofiber. Analysis was performed on 6–10 myofibers for each mouse at each time point. *n* = 3 mice in each group. *p*‐Values were determined by unpaired Student's *t*‐test. *p < 0.05, **p < 0.01, ***p < 0.001. Data are presented as mean ± SD.

Muscle regeneration starts when satellite cells proliferate upon injury.^17^ We stained and quantified PAX7 and Ki67 in the TA muscles of CRY2^flox/flox^ and CRY2^scko^ mice at day 5 after BaCl_2_ injury. The data showed more Ki67 ^+^cells in PAX7^+^ cells in the CRY2^scko^ versus CRY2^flox/flox^ mice, suggesting that deletion of CRY2 led to increased satellite cell proliferation (Figure 4A,B). To confirm these findings, we immunostained PAX7 or MyoD in single myofibers (Figure 4C). Freshly isolated myofibers from CRY2^scko^ mice exhibited an increased number of PAX7^+^/MyoD*^−^* cells (Figure 4D). The myoblasts formed clusters in cultured myofibers (Figure 4C). The number and percentage of PAX7^+^/MyoD^+^ cells per cluster is increased in the CRY2^scko^ compared with CRY2^flox/flox^ mice (Figure 4E,F). There is more Ki67^+^/PAX7^+^ cells on single fibers from CRY2^scko^ mice compared with CRY2^flox/flox^ mice (Figure 4G,H). To understand what happens to the satellite cell pool in older CRY2‐KO mice, we isolated single fibers from 8‐month‐old CRY2^flox/flox^ and CRY2^scko^ mice and immunostained for PAX7 and Ki67. The result showed more PAX7‐positive cells on the single fiber of the CRY2^scko^ mice (Figure 4I,J). Collectively, these findings suggest that deletion of CRY2 enhances satellite cell activation and proliferation.

**FIGURE 5:**
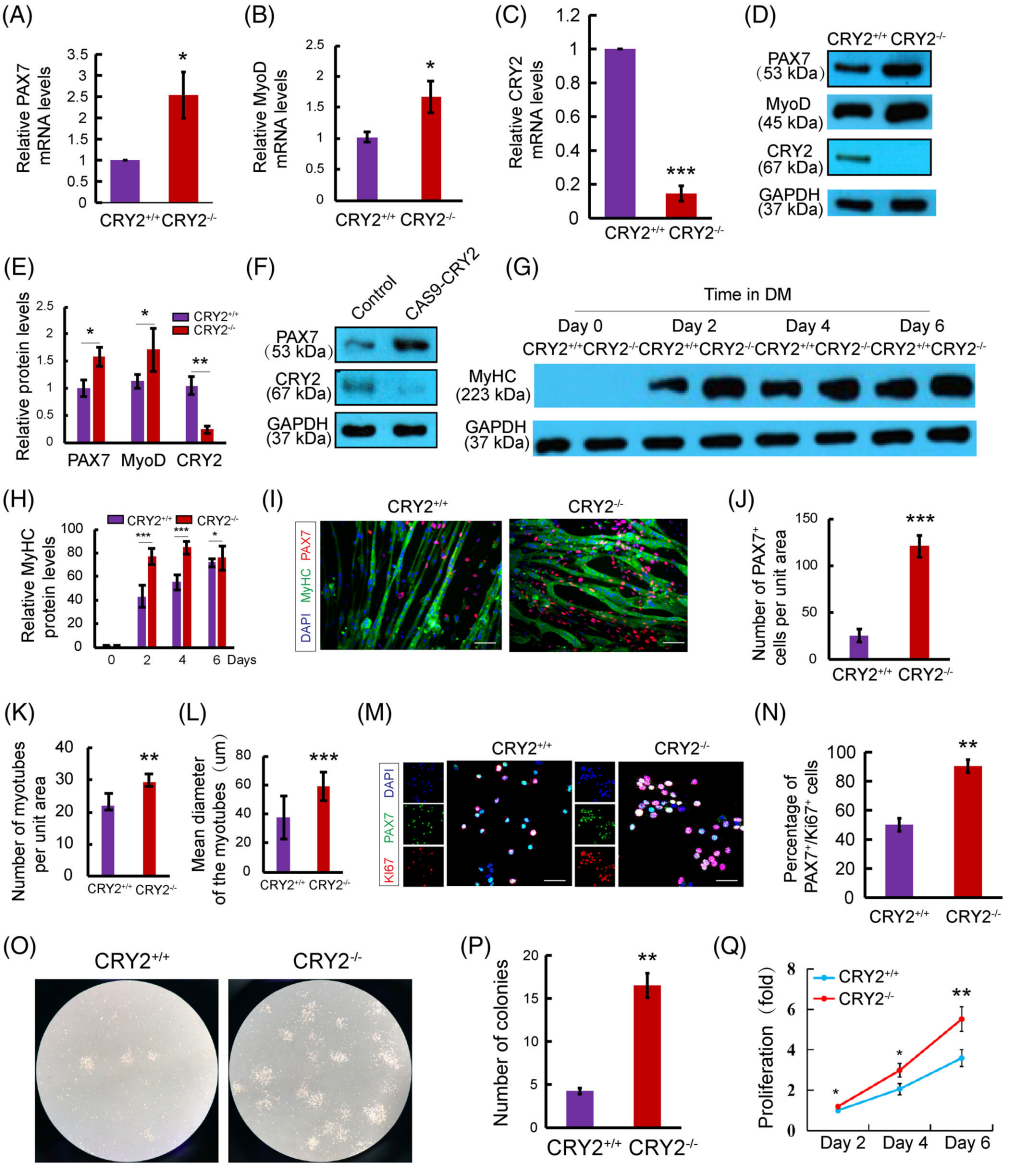
CRY2 regulates satellite cell proliferation and differentiation. (A–C) Real‐time quantitative PCR (RT‐qPCR) analysis of mRNA levels of PAX7, MyoD, and CRY2 in *CRY2^+/+^* and *CRY2^−/−^* primary myoblasts. (D) Western blot analysis of protein levels of PAX7, MyoD, and CRY2 in *CRY2^+/+^* and *CRY2^−/^* ^−^ primary myoblasts. (E) Densitometric quantification of relative protein levels of PAX7, MyoD, and CRY2. (F) Western blot analysis of PAX7 and CRY2 protein levels in primary myoblasts transduced with the empty vector or Cas9‐CRY2 lentiviral construct. (G) Western blot analysis of myosin heavy chain (MHC) protein levels during a 6‐day period of differentiation. (H) Densitometric quantification of relative protein levels of MHC. (I) Immunostaining of MHC (green) and PAX7 (red) in *CRY2^+/+^* and *CRY2^−/−^* primary myoblasts cultured in differentiation medium for 6 days. The scale bar represents 100 μm. (J) Quantification of the number of PAX7^+^ cells per field (0.08 mm^2^). (K and L) Quantification of the number and diameter of myotubes for (I). (M) Immunostaining of PAX7 (green) and Ki67 (red) in *CRY2^+/+^* and *CRY2^−/−^* primary myoblasts cultured for 3 days. Scale bar represents 50 μm. (N) The percentage of PAX7^+^/Ki67^+^ cells. (O) Colony‐forming assay for freshly isolated *CRY2^+/+^* and *CRY2^−/−^* primary myoblasts. (P) Quantification of the number of satellite cell colonies after 5 days in culture. (Q) Cell proliferation was performed by plating *CRY2^+/+^* and *CRY2^−/−^* primary myoblasts at the same density and cultured for 6 days. The cells were enumerated every 2 days. *n* = 3 in each group. *p*‐Values were determined by unpaired Student's *t*‐test. *p < 0.05, **p < 0.01, ***p < 0.001. Data are presented as mean ± SD.

To dissect the causal relationship between CRY2 deletion and PAX7 expression, we compared the expression of PAX7 in *CRY2^+/+^* and *CRY2^−/−^* myoblasts by RT‐qPCR and Western blot (Figure 5) using primary myoblast isolated by FACS sorting (Figure S3A). Loss of CRY2 was verified at both mRNA and protein levels (Figure 5C–E). *CRY2^−/−^* myoblasts exhibited increased PAX7 and MyoD expression compared with *CRY2^+/+^* myoblasts (Figure 5A,B,D, E). We deleted CRY2 in myoblasts using the Cas9 technique to confirm these findings. CRY2‐depleted myoblasts showed an increased protein level of PAX7 compared with the control cells (Figure 5F). Increased myoblast proliferation will make cells challenging to exit cell cycle and go for differentiation, and most of the proliferating myoblasts are MyoD positive. We performed co‐staining and quantification between PAX7 and MyoD in proliferating CRY2^+/+^ and CRY2^−/−^ primary myoblasts and found that CRY2^−/−^ contained more PAX7^+^ and MyoD^+^ cells (Figure S3B,C). This result indicates that loss of CRY2 promotes myoblast proliferation. The differentiation potential of myoblasts was enhanced by the loss of CRY2 evidenced by increased expression of MyHC and myotube formation in *CRY2^−/−^* cells versus *CRY2^+/+^* cells (Figure 5G–I, K,L). Interestingly, immunostaining revealed an increased number of mononucleated PAX7^+^ cells associated with myotubes formed by *CRY2^−/−^* cells compared with *CRY2^+/+^* cells (Figure 5J), suggesting that more reserve cells were produced in the absence of CRY2. Consistent with these findings, the number of PAX7^+^/Ki67^+^ cells is increased in *CRY2^−/−^* cultures (Figure 5M,N). The colony‐forming assay revealed larger colonies formed by *CRY2^−/−^* versus *CRY2^+/+^* cells (Figure 5O,P). Enumeration of satellite cells during 6 days showed an increased number of satellite cell in *CRY2^−/−^* cells (Figure 5Q). These data indicate that CRY2 deletion enhances the proliferation of satellite cells.

### 2.3 CRY2 regulates PAX7 expression and satellite cell proliferation through MAPK/ETS1 pathway

**FIGURE 6:**
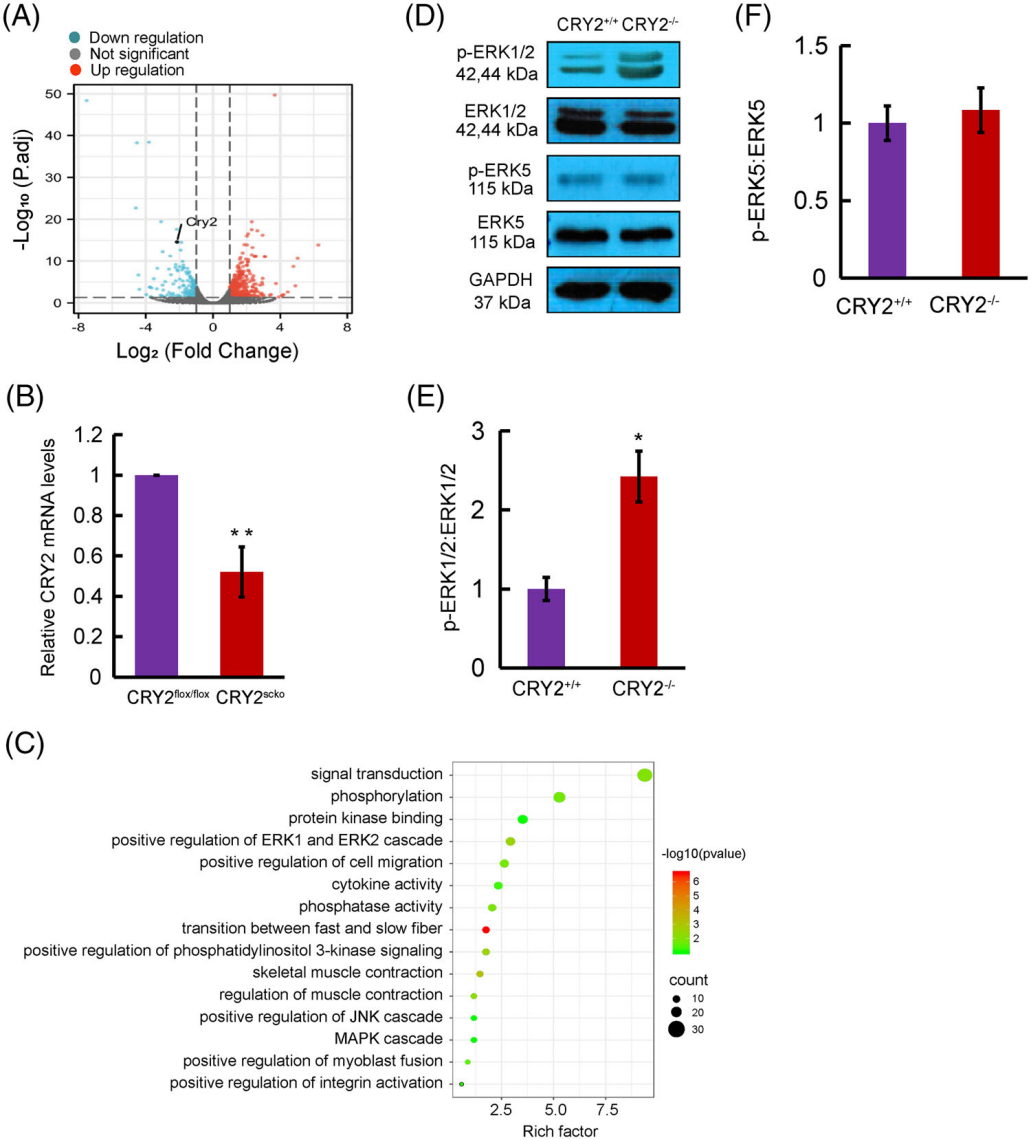
CRY2 deficiency leads to the activation of ERK/1/2 signaling pathway. (A) The volcano diagrams of differentially regulated genes in the gastrocnemius muscles from CRY2^flox/flox^ and CRY2^scko^ mice were made using the package R ggplot2. CRY2 is among the downregulated genes as shown in the diagram. (B) Real‐time quantitative PCR (RT‐qPCR) analysis of mRNA levels of CRY2 in gastrocnemius muscles in from CRY2^flox/flox^ and CRY2^scko^ mice. *n* = 3 independent experiments. (C) Gene ontology (GO) analyses of upregulated differentially expressed genes in the gastrocnemius muscles from CRY2^flox/flox^ and CRY2^scko^ mice were performed using DAVID bioinformatics resources. (D) Western blot analysis of phosphorylated and total ERK1/2, ERK5 in *CRY2^+/+^* and *CRY2^−/−^* primary myoblasts. (E) The ratio of phospho‐ERK1/2 and total ERK1/2 in *CRY2^+/+^* and *CRY2^−/−^* primary myoblasts. (F) Ratio of phospho‐ERK5 and total ERK5. *n* = 3 in each group. *p*‐Values were determined by unpaired Student's *t*‐test.* *p* < 0.05, **p < 0.01. Data are presented as mean ± SD.

To determine how CRY2 deletion affects satellite cell function, we performed RNAseq on mRNAs from gastrocnemius muscles of CRY2^scko^ mice and CRY2^flox/flox^ mice. In CRY2^scko^ muscle, 374 genes showed an increased expression, whereas 155 genes displayed a decreased expression (Figure 6A). RNAseq also revealed a 4.41‐fold reduction of CRY2 in the CRY2^scko^ muscle compared with CRY2^flox/flox^ muscle, which was confirmed by RT‐qPCR (Figure 6B).

Kyoto Encyclopedia of Genes and Genomes (KEGG) and gene ontology (GO) analyses^18^, ^19^ were performed to find out signaling pathways that were differentially regulated by CRY2. GO analysis of upregulated genes revealed positive regulation of ERK1/2 cascade (Figure 6C), suggesting that deletion of CRY2 led to the activation of the MAPK signaling pathway, which control PAX7 expression.^20^ We, therefore, evaluated whether ERK1/2 can be phosphorylated in the absence of CRY2. *CRY2^−/−^* cells exhibited increased phospho‐ERK1/2 compared with *CRY2^+/+^* cells (Figure 6D,E), suggesting that deletion of CRY2 led to activation of ERK1/2. As an activation of the ERK5 MAPK pathway induces myogenic differentiation,^20^, ^21^ we asked whether ERK5 could mediate CRY2 signaling in satellite cells. However, p‐ERK5 did not show a significant difference between *CRY2^−/−^* cells and *CRY2^+/+^* cells (Figure 6D,F).

**FIGURE 7:**
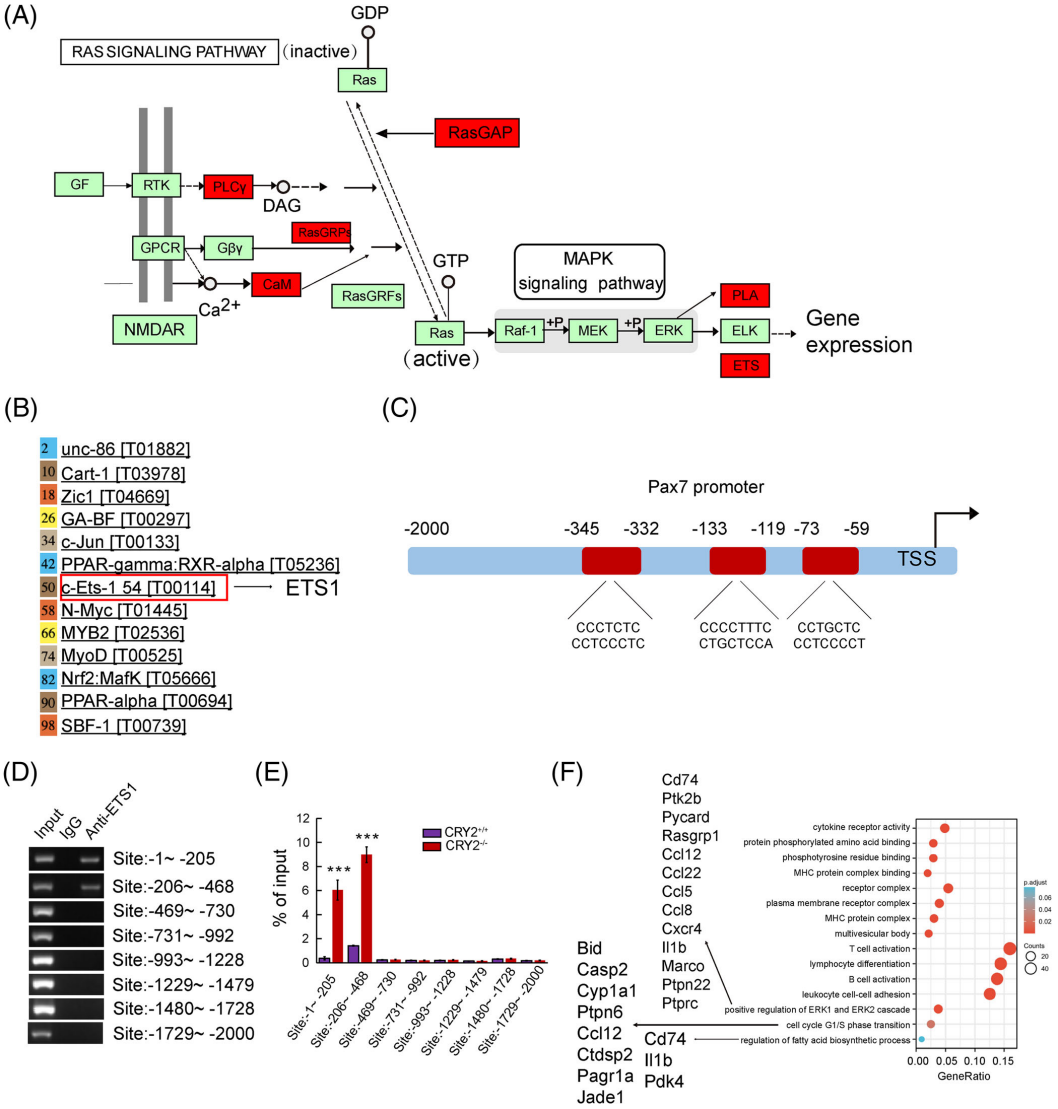
Role of ETS1 in regulating PAX7 expression. (A) Kyoto Encyclopedia of Genes and Genomes (KEGG) analysis using DAVID bioinformatics resources shows that ERK is an upstream regulator for ETS1. (B) Bioinformatics analysis of transcription factors that can potentially bind to PAX7 promoter was predicted by PROMO database. (C) Prediction of ETS1‐binding sites on PAX7 promoter by JASPAR. (D) Primary myoblasts were processed for ChIP assay. The semiquantitative RT‐PCR showed that ETS1 was enriched at the indicated sites of the PAX7 promoter. The numbers represent the location of the consensus sequence upstream of the first ATG of the Pax7 gene. (E) Real‐time quantitative PCR (RT‐qPCR) analysis of ChIP product to determine the percentage of ETS1 input enrichment at specific sites in the PAX7 promoter in *CRY2^+/+^* and *CRY2^−/−^* primary myoblasts. (F) Gene ontology (GO) analyses of upregulated differentially expressed genes in the gastrocnemius muscles from CRY2^scko^ mice and CRY2 ^flox/flox^ mice were made with DAVID bioinformatics resources. *p*‐Values were determined by unpaired Student's *t*‐test.*** *p* < 0.001. *n* = 3 in each group. Mice used to start the experiment were pooled at 6–8 weeks of age. Data are presented as mean ± SD. I, injured; U, uninjured

**FIGURE 8:**
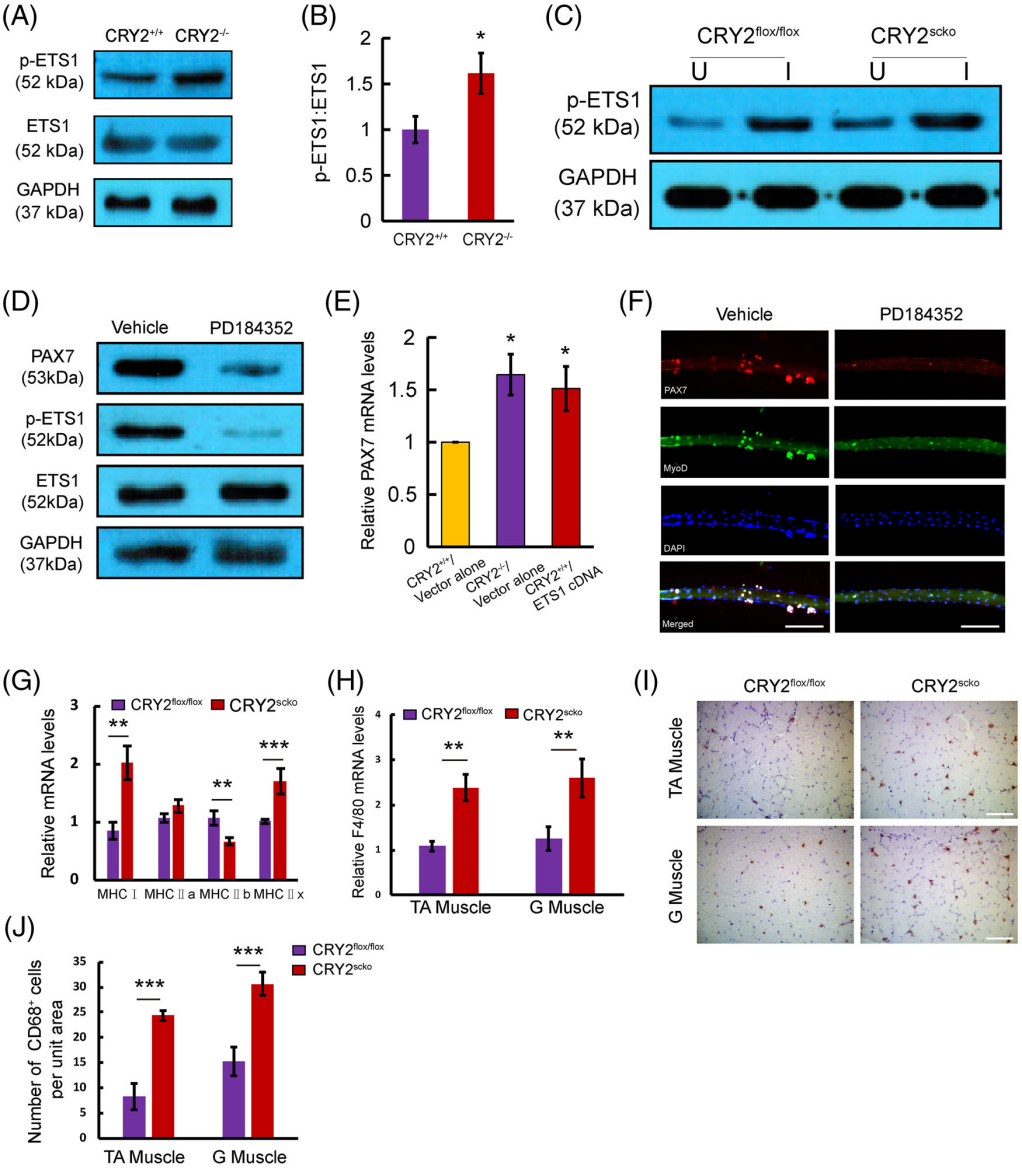
Role of ERK1/2/ETS1 signaling pathway in regulating the self‐renewal of primary myoblasts. (A) Western blot analysis of phosphorylated and total ETS1 in *CRY2^+/+^* and *CRY2^−/−^* primary myoblasts. (B) The ratio of phospho‐ETS1 and total ETS1. *p*‐Values were determined by unpaired Student's *t*‐test. (C) Western blot analysis of p‐ETS1 in uninjured and injured TA muscles of CRY2^flox/flox^ and CRY2^scko^ mice. (D) Western blot analysis of PAX7, phospho‐, and total ETS1 protein. Primary myoblasts were treated with 2 μM PD184352 or vehicle for 4 h. (E) Real‐time quantitative PCR (RT‐qPCR) analysis of mRNA levels of PAX7 in CRY2 ^+/+^ vector alone, CRY2^−/−^ vector alone and CRY2^+/+^ cells overexpressing ETS1 cDNA. (F) Single myofibers isolated from extensor digitorum longus (EDL) muscle of WT mice were cultured in a satellite cell growth medium in the presence of ERK1/2 inhibitor for 72 h. The single myofibers were stained for PAX7 (red) and MyoD (green). Nuclei were identified by staining with DAPI (blue). Scale bars: 100 μm. Analysis was performed using 3–6 myofibers for each mouse in each group. (G) Relative mRNA levels of MHCI, MHCIIa, MHCIIb, and MHCIIx. (H) Real‐time quantitative PCR (RT‐qPCR) analysis of mRNA levels of F4/80 in TA and gastrocnemius muscles from CRY2^flox/flox^ and CRY2^scko^ mice. (I) Immunohistochemistry of CD68 in TA and gastrocnemius muscles from CRY2^flox/flox^ and CRY2^scko^ mice. Scale bars: 50 μm. (J) Quantification of the number of CD68^+^ cells per unit area (0.08 mm^2^) for (I). *n* = 3 mice in each group. *p*‐Values were determined by unpaired Student's *t*‐test.* *p* < 0.05, **p < 0.01, ***p < 0.001. Data are presented as mean ± SD.

KEGG analysis of upregulated genes from our RNAseq revealed ETS1 as a target downstream of MEK and ERK pathways (Figure 7A). In silico analysis showed that ETS1 has the potential to bind to the PAX7 promoter (Figure 7B). JASPAR database revealed three binding sites for ETS1 (Figure 7C). Enrichment of ETS1 was found in the PAX7 promoter at two consensus sites, as shown by the ChIP assay (Figure 7D). Furthermore, *CRY2^−/−^* myoblasts exhibited an increased ETS1 enrichment at the PAX7 promoter compared with *CRY2^+/+^* myoblasts (Figure 7E). These findings are in‐line with the previous studies showing that ERK1/2 could phosphorylate and activate ETS1.^22^, ^23^ We, therefore, asked whether the level of phospho‐ETS1 is increased in the absence of CRY2. Western blot analysis revealed that p‐ETS1 was increased in *CRY2^−/−^* versus *CRY2^+/+^* cells (Figure 8A,B). Notably, the injured CRY2^scko^ TA muscle exhibited increased levels of p‐ETS1 compared with CRY2^flox/flox^ muscle (Figure 8C). To ascertain the regulation of MAPK on the expression of p‐ETS1 and PAX7, primary myoblasts were treated with PD184352, which is a specific inhibitor for ERK1/2 pathway. Cells treated with PD184352 displayed decreased expression of PAX7 and p‐ETS1 (Figure 8D). We then asked whether an overexpression of ETS1 in *CRY2^+/+^* cells could upregulate PAX7 expression. RT‐qPCR showed that PAX7 mRNA level was increased in *CRY2^+/+^* cells overexpressing ETS1 compared to cells transduced with empty vector (Figure 8E). EdU staining and differentiation assay of primary myoblasts revealed that ETS1 overexpression promotes cell proliferation (Figure S4A,B) and differentiation (Figure S4C). Immunostaining revealed a decreased number of cell clusters and the number of PAX7^+^/MyoD^+^ cells when myofibers were treated with PD184352 (Figure 8F), confirming that ERK1/2 is involved in promoting satellite cell activation and proliferation in the absence of CRY2. We analyzed the mRNA expression of different fiber types in the gastrocnemius muscle of CRY2^flox/flox^ and CRY2^scko^ mice. The data showed that type I and type IIx were significantly upregulated, whereas type IIb was significantly downregulated resulting from CRY2 deletion (Figure 8G). The molecules that a positive regulation of ERK1 and ERK2 cascade includes are Cd74, Ptk2b, Pycard, Rasgrp1, Ccl12, Ccl22, Ccl5, Ccl8, Cxcr4, Il1b, Marco, Ptpn22, and Ptprc (Figure 7F). GO enrichment analysis further reveals that G1/S transition of the cell cycle and regulation of fatty acid biosynthetic process is increased (Figure 7F). The molecules that positively regulate ERK1 and ERK2 are secreted by immune cells (mainly from macrophages) within the muscle, which directly suggests that higher inflammation state in the hemostasis of the CRY2^scko^ muscle. We, therefore, performed RT‐qPCR and immunostaining to quantify macrophages from the gastrocnemius and TA muscles. RT‐qPCR analysis showed that the mRNA level of macrophage marker F4/80 is increased in the gastrocnemius and TA muscles from CRY2^scko^ mice compared with CRY2^flox/flox^ mice (Figure 8H). Immunostaining of CD68 revealed more macrophages in the gastrocnemius and TA muscles from the CRY2^scko^ mice versus CRY2^flox/flox^ mice (Figure 8I,J). These results indicate that CRY2 deletion promotes the recruitment of macrophages to the skeletal muscle, which might contribute to the activation of satellite cells.

### 2.4 Deletion of CRY2 improves the survival rate of satellite cells in ischemic muscles

We further addressed whether CRY2 deletion enhances satellite cell survival and proliferation by studying the engraftment of *CRY2^−/−^* and *CRY2^+/+^* cells in ischemic muscle. Satellite cells from CRY2^scko^ and CRY2^flox/flox^ mice were transduced with a lentiviral vector expressing GFP and delivered into the ischemic TA muscles of WT mice. TTC staining confirmed that ischemia and reperfusion injury induces muscle necrosis (Figure S5A,B). Fluorescent microscopy revealed more GFP^+^ cells in muscles injected with *CRY2^−/−^* cells as compared with that of the *CRY2^+/+^* cells on day 7 postinjection (Figure S5C). Most transplanted GFP^+^/*CRY2^−/−^* cells also express PAX7 (Figure S5C,D), indicating that the deletion of CRY2 improves satellite cell engraftment in vivo.

The increased survival rate of *CRY2^−/−^* cells in ischemic muscles suggests that these cells may be more resistant to apoptotic stimuli. To test this hypothesis, we performed TUNEL staining in TA muscles of CRY2^scko^ and CRY2^flox/flox^ mice 7 days after ischemia‐reperfusion (IR) injury. More than 30% of PAX7^+^ satellite cells from CRY2^flox/flox^ mice showed positive TUNEL staining, whereas only about 10% of PAX7^+^ satellite cells in CRY2^scko^ mice were apoptotic (Figure S6A,B).

Collectively, we conclude that CRY2 is downregulated upon injury, and loss of CRY2 allows skeletal muscle to rapidly replenish the stem cell pool through the upregulation of PAX7, leading to enhanced satellite cell function during muscle repair.

## 3 DISCUSSION

The circadian clock regulates skeletal muscle function. However, the role of CRY2 in muscle regeneration remains controversial. The present study demonstrates that satellite cell‐specific deletion of CRY2 promotes muscle repair. Single myofiber analysis revealed an increased number of PAX7^+^/MyoD*^−^* satellite cells in uncultured myofibers of CRY2^scko^ mice. CRY2‐deficient satellite cells displayed increased PAX7 expression and proliferation compared with *CRY2^+/+^* cells. Our data indicate that CRY2 downregulation is necessary for satellite cell proliferation during regenerative myogenesis.

CRY2‐depleted satellite cells showed an increased protein level of PAX7 compared with the control cells. Immunostaining revealed more proliferating PAX7^+^/Ki67^+^ satellite cells in *CRY2^−/−^* cultures. Therefore, deletion of CRY2 leads to enhanced proliferation of satellite cells engaged in differentiation.

It has been shown that the activation of the ERK1/2 signaling pathway can lead to increased PAX7 expression.^20^, ^24^ Our data revealed an increased expression of p‐ERK1/2 in *CRY2^−/−^* satellite cells, suggesting that the increased PAX7 expression in these cells may be mediated by a specific MAPK signaling pathway.

Our RNAseq data revealed transcription factor ETS1 as a downstream target of ERK1/2. ETS1 binds to PAX7 promoter at two consensus sites as shown in ChIP assay. The fold enrichment of ETS1 was significantly increased in satellite cells from CRY2^scko^ mice versus control. These data suggest that the loss of CRY2 enhanced the binding of ETS1 to the PAX7 promoter. We demonstrated that it was reduced in the presence of ERK1/2 inhibitor. Furthermore, p‐ETS1 levels were increased in *CRY2^−/−^* cells. These results confirm that CRY2 deletion resulted in increased phosphorylation of ERK1/2, which phosphorylates ETS1, and induces PAX7 transcription. Our findings align with previous reports that an inhibition of MAPK reduced the phosphorylation and activation of ETS1 in human cholangiocytes^23^ and breast cancer cells^25^ and that ERK2‐mediated phosphorylation of ETS1 enhances its binding to CREB‐binding protein.^22^ We further demonstrated an increased PAX7 mRNA expression in *CRY2^+/+^* cells overexpressing ETS1 versus control cells. The result is consistent with our findings that CRY2 deletion increased the expression of p‐ETS1 and PAX7, leading to increased satellite cell proliferation.

CRY2 is also expressed in skeletal muscle cells,^26^, ^27^, ^28^ leukocyte,^29^ endothelial cells,^30^ and vascular smooth muscle cells.^31^ CRY2 gene expression varies depending on the time of the day and is affected by feeding^32^ and ambient temperature.^28^ CRY2 expression is also altered by metabolic processes^33^ and obesity.^27^ Upon muscle injury, leukocytes are responsible for removing dead fibers, which is the first phase of muscle regeneration. However, chronic leukocyte infiltration can induce secondary injury by producing inflammatory cytokines. Thus, further understanding how CRY2 regulates leukocyte infiltration will provide useful information for muscle regeneration. Endothelial and smooth muscle cells participate in angiogenesis, which is important during muscle regeneration. Future research on the role of CRY2 in regulating the proliferation of endothelial cells and smooth muscle cells could enhance our understanding of angiogenesis.

Our study uncovered a new role of CRY2 in regulating satellite cell proliferation and differentiation. CRY2 deletion enhances satellite cell self‐renewal by upregulating PAX7 via the ERK1/2/ETS1 pathway. Downregulation of CRY2 is necessary to maintain satellite cell self‐renewal to sustain the regenerative potential of skeletal muscle for repetitive injury. These findings pave the way for new therapeutic approaches to promote muscle regeneration.

*Limitations of this study*: We demonstrated that satellite cell‐specific CRY2 deletion promotes muscle regeneration, which could be the result of an increased number of satellite cells during development. To address this issue, we should use PAX7‐CreER model to induce PAX7 after birth in the future. It is known that slow myofibers generally have more satellite cells than fast‐type myofibers.^34^ The mechanisms underlying this phenomenon remain largely unknown. It may be due to the heterogeneity of the satellite cells residing in different types of myofibers. Future studies are required to determine whether and how CRY2 regulates satellite subpopulations in different types of myofibers.

Using a macrophage and satellite cell coculture model, Massimino et al. showed that macrophage‐derived factors stimulate the proliferation and differentiation of satellite cells.^35^ Recent studies showed that macrophages play critical roles in activating satellite cells via TNF‐α,^36^ IL‐6,^37^ IL‐1β,^38^ and glutamine.^39^ In an endotoxin‐induced liver injury model, Wang et al. showed that the deletion of the circadian clock gene Per1 promotes the infiltration of macrophages to the liver by enhancing hepatic Ccr2 through the PPAR‐γ pathway.^40^ Future studies are required to determine whether loss of CRY2 could attract macrophages to skeletal muscle by enhancing Ccr2 expression.

## 4 MATERIALS AND METHODS

### 4.1 Animals

C57BL6/J mice were from Shanghai SLAC Laboratory Animal Co,. Ltd (Shanghai, China). CRY2^scko^ mice were produced by mating Pax7‐Cre mice (Stock Number: 010530, Pax7tm1 (cre) Mrc/J; The Jackson Laboratory, Bar Harbor, ME) with CRY2^flox/flox^ mice (CAM‐SU Genomic Resource Center of Soochow University, Suzhou, China). The genotype of mice was confirmed by PCR and DNA sequencing using genomic DNA. The deletion of CRY2 was verified by RT‐qPCR and Western blot. The mice were males of 6–8 weeks unless otherwise indicated. All animal experiments were performed according to the ARRIVE guidelines and National Institutes of Health guide for the care and use of laboratory animals (NIH Publications No. 8023, revised 1978). The mice were randomized, and the investigator was blinded to the group allocation during the experiments and when assessing the outcome.

### 4.2 BaCl_2_ injury model

The TA muscles CRY2^flox/flox^ and CRY2^scko^ mice were injected with 100 μl of 1.2% BaCl_2_ (Cat# 342920, Sigma‐Aldrich, St. Louis, MO). The contralateral uninjured TA muscle was used as control.

### 4.3 Histological analysis

The muscles were frozen in liquid nitrogen‐cooled isopentane. The frozen muscles were sectioned using a microtome cryostat (Leica CM 1950, Leica, Wetzlar, Germany). Ten sections were cut, spanning the entire muscle, and stained with H&E (Cat#C0105, Beyotime, Shanghai, China), and the number of CNF and CSA of myofibers was analyzed under a Leica DM2000 (Leica) microscope. TTC staining was used to assess muscle necrosis using a commercial kit (Cat#G3005, Solarbio, Beijing, China).

### 4.4 Isolation of primary myoblasts

Primary myoblasts were isolated from TA muscle, as we have described previously.^41^ Briefly, TA muscle was minced and digested by Dispase type II, and collagenase D followed by filtration through a 70 μm nylon mesh (Fisher Scientific, 22363548) to remove debris. The filtrate was cultured in myoblast growth medium (Shanxi Anning Yunsheng Biotechnology Co., Ltd, 60071‐1) and then pre‐plated twice to remove non‐myogenic cells.

### 4.5 Primary myoblast isolation by FACS

The tibialis anterior and gastrocnemius muscles were removed from 6 to 8‐week mice and digested as mentioned above. After terminating digestion, the cells were resuspended in ice‐cold PBS at 300*g*, centrifuged for 5 min, washed twice, and resuspended in ice‐cold PBS. The following antibodies were added into 200 μl of the cell suspension: CD31‐PE (Cat#102408, BD BioLegend), CD45‐PE (Cat#561087, BD Biosciences), Sca‐1‐PE (Cat#553108, BD Biosciences), CD11b‐PE (Cat#557397, BD Biosciences), and Integrin α7 Alexa Fluor 647‐conjugated Antibody (Cat#FAB3518R, R&D Systems). After incubating on ice for 30 min, the cells were centrifuged at 300*g* for 5 min and washed twice with ice‐cold PBS. The cells were resuspended with 500 ul PBS containing 2%PS and 2%FBS, filtered through a 70 micrometer filter (Fisher Scientific, 22363548), and sorted on a flow cell sorter (Flow Cytometer, FACSAria III). Satellite cells were gated for α7‐Integrin after eliminating all CD45, CD31, CD11b, and Sca‐1 positive cells from all mononuclear cells.

### 4.6 Analysis of the expression of circadian genes

CRY2^flox/flox^ and CRY2^scko^ mice were maintained in a 12‐h light and 12‐h dark cycles (8 a.m.–8 p.m. light and 8 p.m. –8 a.m. dark) environment for 2 weeks before the experiments were started. Zeitgeber time 0 (ZT0) and ZT12 refer to 8 a.m. and 8 p.m., respectively. TA muscles were taken every 4 h starting at ZT2. The RT‐qPCR primer sequences are shown in Table S1.

### 4.7 Colony assay

Freshly isolated satellite cells were seeded in a 35 mm dish (Cat# 150460, Thermo Fisher Scientific) to form colonies, which were counted 5 days after culture under a microscope.

### 4.8 Single myofiber isolation

Single myofiber was prepared from the extensor digitorum longus (EDL) muscle as described previously.^41^ Briefly, the EDL muscles were digested with collagenase type I, followed by culturing the myofibers on 10% matrigel‐coated dishes.

### 4.9 Immunofluorescence analysis of myogenic markers

Frozen muscle sections, as well as myofibers, and primary myoblasts were fixed and then incubated with primary antibodies. CRY2 (1:100, SAB1300079) and Laminin (1:500, Cat# L9393) antibodies were purchased from Sigma. Antibodies against PAX7, eMyHC, MyHC, MyoD, CD68 (1:200, Cat# 97778S), and anti‐Ki67 were from Cell Signaling Technology (1:50, Cat# 12075, Danvers, MA). The secondary antibodies have been described in detail by our lab previously.^41^ The images were captured by a confocal microscope (Olympus Fluoview FV3000, Olympus) and analyzed with Olympus FV31S‐SW software.

### 4.10 RT‐qPCR

RNA extraction, reverse transcription, and RT‐qPCR were performed as we have described previously.^41^ Briefly, total RNA was reverse transcribed into cDNA followed by RT‐qPCR using SYBR Premix Ex Taq (Cat# RR420B, Takara, Osaka, Japan) and the QuantStudio 6 Flex Real‐Time PCR System (Thermo Fisher Scientific). The RT‐qPCR primer sequences are shown in Table S2.

### 4.11 RNA sequencing (RNAseq) and bioinformatic analysis

#### RNA‐seq was performed by Shanghai Biotechnology Corporation (Shanghai, China). Gastrocnemius muscles from CRY2^scko^ mice and CRY2^flox/flox^ mice were used to extract RNA, generating cDNA library, followed by sequencing on an Illumina system (Illumina) as we have described previously.^41^

### 4.12 Western blot

Proteins extracted from cells or muscles were separated by SDS–PAGE and then blotted to PVDF membranes as described previously.^41^ CRY2 antibody was from Sigma (1:1000, Cat# SAB1300079). ETS1 (1:1000, Cat# 14069S) was from Cell Signaling Technology. Antibodies against phospho‐ETS1 (1:1000, Cat# SAB4503912) were from Sigma. Antibodies against MyoD, PAX7, GAPDH, phospho‐ERK1/2, ERK1/2, phospho‐ERK5, ERK5, and HRP‐conjugated secondary antibodies have been described in detail previously.^41^

### 4.13 Cell proliferation assay

Primary myoblasts were grown in 2% rat tail collagen‐coated culture plates (Cat# A10483‐01, Invitrogen). After fixation, the cells were incubated with anti‐Ki67 (1:50, Cat# 12075, Cell Signaling Technology) and anti‐PAX7 (1:10, clone PAX7‐s, DSHB).^41^

### 4.14 Myogenic differentiation

Primary myoblasts were seeded on 6‐well cell culture plates coated with 2% rat tail collagen. Differentiation was triggered by adding 2% horse serum in DMEM (Cat# 16050122, Gibco).

### 4.15 Construction of lentivirus overexpressing CRY2 or ETS1

CRY2 or ETS1 cDNA was cloned into LV5 plasmid (Puromycin resistance, GenePharma) with BamH1 and Sph1 or Not1 restriction enzymes, and packaging plasmids ΔR8.74, VSV‐G, and Rev were co‐transfected into 293T cells as described previously.^41^ The packaging plasmids were provided by Dr. Yun Zhao, Soochow University. The constructs were confirmed by DNA sequencing using the primers in Table S3.

### 4.16 Construction of CRY2 knockout lentivirus using CRISPR/Cas9 technique

CRY2‐sgRNA was cloned into a V2 plasmid with BsmB1/Esp3I restriction enzymes (Cat# ER0452, Thermo Fisher Scientific). Lentiviral particles were produced by transfecting the 293T cells with the V2 plasmid containing CRY2‐sgRNA and packaging plasmids ΔR8.74, VSV‐G, and Rev. The sequence of the constructed Cas9‐CRY2 plasmids was confirmed by DNA sequencing using the primers in Table S3.

### 4.17 Chromatin immunoprecipitation

The ChIP assay was carried out using the ChIP‐IT Express Kit (Cat# 53008, Active motif, Carlsbad, CA) as described previously.^41^ Briefly, primary myoblasts were cross‐linked with 1% formaldehyde, and the cells were lysed to extract the nuclei. After fragmentation by sonication, the DNA samples were precleared with normal IgG and then incubated with the anti‐ETS1 antibody (4 μl per 1 × 10^7^ cells) and protein G Magnetic beads. The precipitated DNA was amplified by PCR. PCR primers were designed to cover the PAX7 promoter 2000 bp upstream of the transcription start site primers are shown in Table S4.

### 4.18 EdU staining

Primary myoblasts were labeled with EdU for 2 h (Cell Proliferation Kit, Cat# C0075S, Beyotime) followed by fixation and permeabilization with 0.2% Triton X‐100.

### 4.19 Ischemia‐reperfusion (IR) injury and primary myoblast transplantation

Under anesthesia, IR injury was induced by placing an orthodontic rubber band at the hip joint. After 3 h of ischemia and 24 h of reperfusion, TA muscle was injected with 1 × 10^6^ GFP^+^ *CRY2^+/+^* or GFP^+^ *CRY2^−/−^* primary myoblasts transduced with GFP^+^LV5 lentivirus. TA muscle was isolated for histological analysis 7 days after transplantation.

### 4.20 TUNEL staining

Frozen tibial anterior muscle sections were fixed and permeabilized in 0.1% Triton X‐100 for 10 min. After incubation in immunostaining blocking solution for 1 h at room temperature, the sections were incubated with anti‐PAX7 antibody overnight at 4°C. After washing, the sections were incubated with fluorescent secondary antibody for 1 h. The TUNEL Apoptosis Detection Kit was used to detect apoptotic cells (Cat# C1089, Beyotime).

### 4.21 Statistics

Data processing was performed by using ImageJ software and data analysis using Statistical Product Service Solutions (SPSS) software. Data are presented as means ± SD. Student's *t*‐test was used to determine the significance of the differences between the two groups. *p* < 0.05 was considered statistically significant. Sample‐size determinations were based on achieving a power of 80% and an experiment‐wise error rate of 5%.

## AUTHOR CONTRIBUTIONS

*Conceptualization; design of the study; and writing*: Yangxin Li, Yao‐Hua Song. *Acquisition of data, or analysis and interpretation of data*: Yingxue Hao, Ting Xue, Song‐Bai Liu, Sha Geng, Xinghong Shi, Panting Qian, Wei He, Jiqing Zheng, Yanfang Li, Jing Lou, Tianze Shi, Ge Wang, Xiaoxiao Wang, Yanli Wang. All authors have read and approved the final manuscript.

## CONFLICT OF INTEREST

The authors declare no conflict of interest.

## ETHICS STATEMENT

All animal protocols were approved by the Institutional Laboratory Animal Care and Use Committee of Soochow University (No. SUDA20220826A01).

## Supporting information



Supporting Information

## Data Availability

The data included in this study are available upon request from the corresponding author.
