# Supplementary material for: Loss of CRY2 promotes regenerative myogenesis by enhancing PAX7 expression and satellite cell proliferation
Source: MedComm (2020). 2023 Jan 9;4(1):e202. doi: 10.1002/mco2.202 (PMC9830134; doi:10.1002/mco2.202)
Supplement: Supplementary file 1 — Supporting Information [file MCO2-4-e202-s001.pdf]

Supplementary materials

Loss of CRY2 promotes regenerative myogenesis by enhancing PAX7 expression and satellite cell proliferation

Running title: Loss of CRY2 promotes regenerative myogenesis

Yingxue Hao[1#], Ting Xue[1#], Song-Bai Liu[2#], Sha Geng[1#], Xinghong Shi[1#], Panting Qian[1], Wei He[1], Jiqing Zheng[1], Yanfang Li[1], Jing Lou[1], Tianze Shi[1], Ge Wang[1], Xiaoxiao Wang[2], Yanli Wang[3], Yangxin Li[3]*, Yao-Hua Song[1]*

Supplemental Figure 1

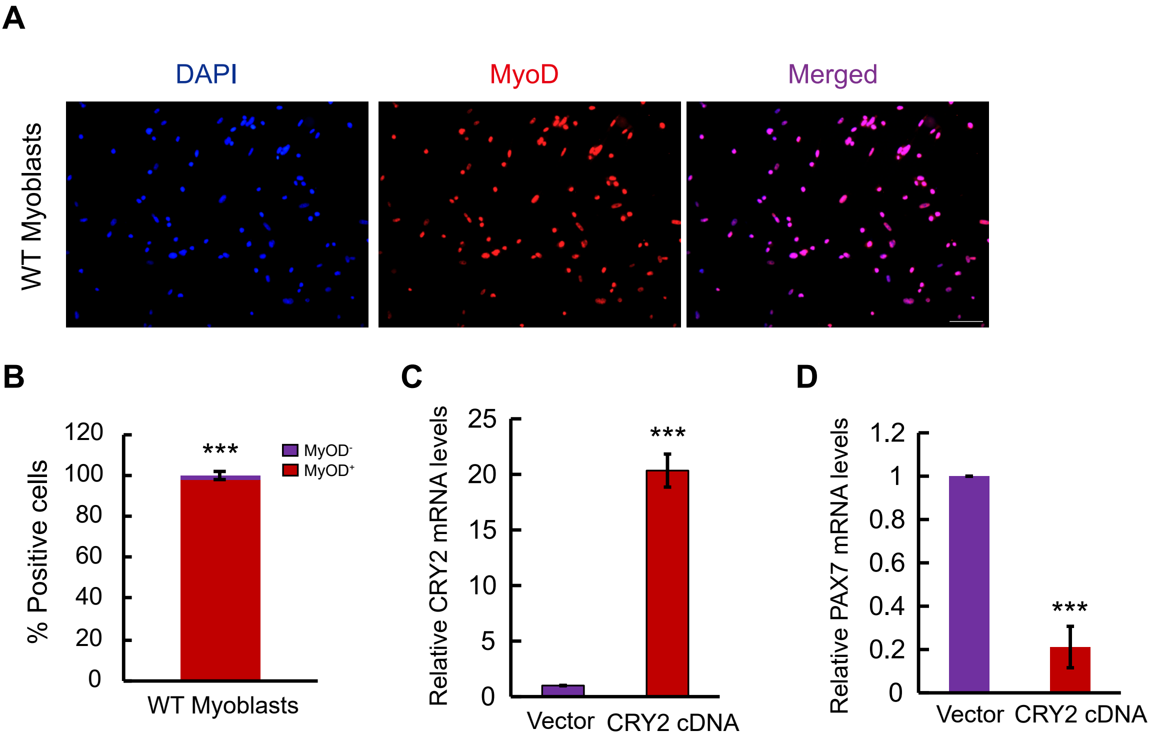

Supplemental Figure 1. Overexpression of CRY2 inhibits PAX7 expression in satellite cells. (A) Immunofluorescence staining of MyoD (red) of primary myoblasts from C57BL/6J (WT). Nuclei were stained with DAPI (blue). Scale bars: 100 μm. (B) Quantification of the percentage of $MyoD^+$cells in primary myoblasts.RT-qPCR analysis of mRNA levels of CRY2 (C) and PAX7 (D) in primary myoblasts transduced with lentivirus overexpressing CRY2 cDNA or empty vector. n = 3 mice in each group. Mice used to start the experiment were pooled at 6 – 8 weeks of age. P values determined by unpaired Student's t-test.***$P<0.001$. Data are presented as mean ± SD.

Supplemental Figure 2

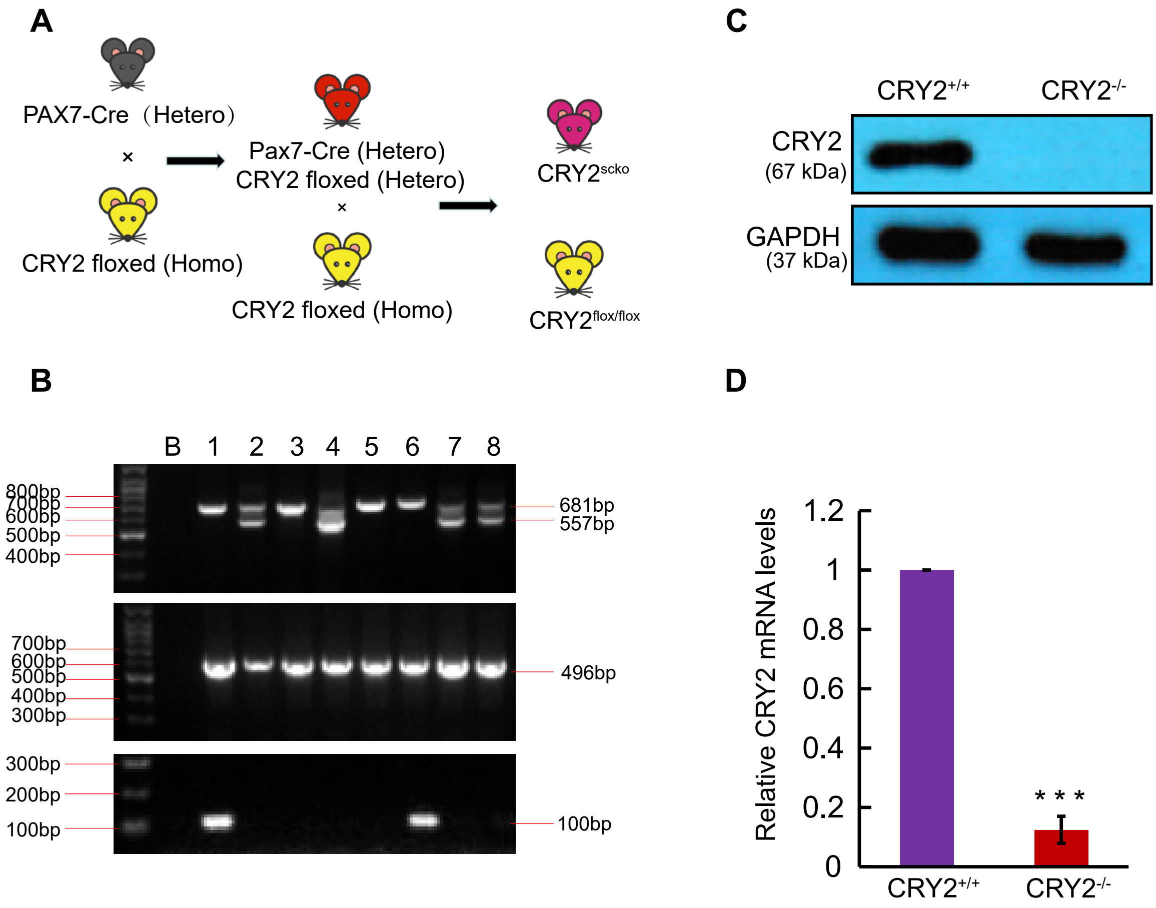

Supplemental Figure 2. Generation of skeletal muscle lineage and satellite cell-specific CRY2 knockout mice. (A) Breeding strategy to obtain $CRY2^{scko}$ and littermate $CRY2^{flox/flox}$ mice. (B)The genotype of $CRY2^{scko}$ and littermate $CRY2^{flox/flox}$ mice was identified by PCR and agarose gel electrophoresis using genomic DNA. The PCR product of $CRY2^{flox/flox}$ homozygous mice is 681 bp. For the Pax7-cre mice, the PCR product of wild-type genotype is 496 bp, mutant PCR product is 100 bp, heterozygote PCR product is 496 and 100 bp. Identification results: mice #1, #6 had 681, 496, and 100 bp bands; therefore, these mice are $CRY2^{scko}$. Mice #3 #5 had 681 and 496 bands; these mice are $CRY2^{flox/flox}$. B: blank, no template DNA control. (C)Western blot analysis of protein level of CRY2 from primary myoblasts of $CRY2^{flox/flox}$ and $CRY2^{scko}$ mice. (D) RT-qPCR analysis of mRNA level of CRY2 from primary myoblasts of $CRY2^{flox/flox}$ and $CRY2^{scko}$ mice. n = 3 mice in each group. P values were determined by unpaired Student's t-test. ****P*<0.001. Data are presented as mean ± SD.

Supplemental Figure 3

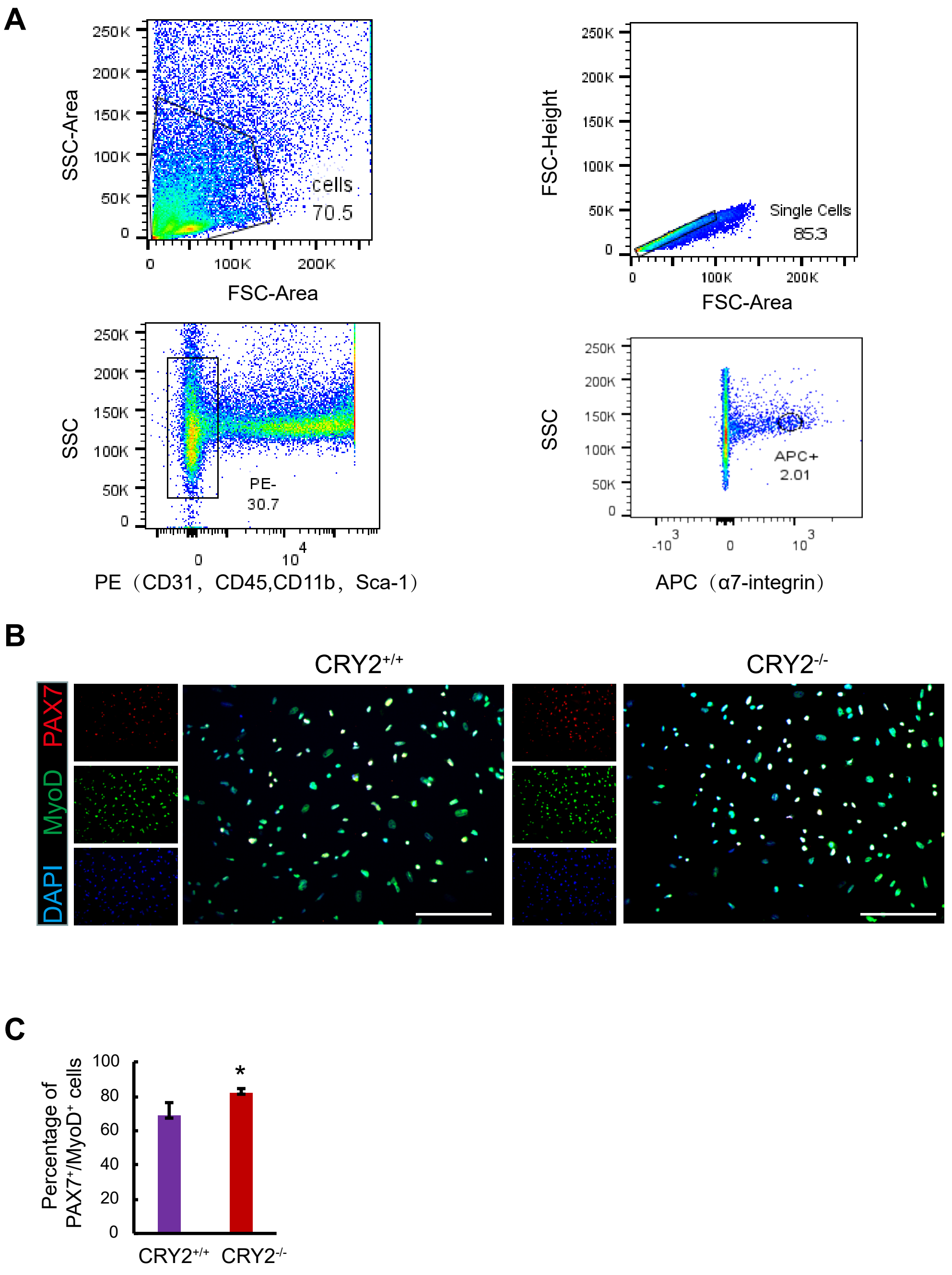

Supplemental Figure 3. Deletion of CRY2 in satellate cells increased the number of PAX7 and MyoD double-positive cells. (A) FACS gating schematic for identification of satellite cells. Satellite cells were gated for α7-Integrin after eliminating all CD45, CD31,CD11b and Sca-1 positive cells from all mononuclear cells. (B) Immunostaining of PAX7 (red) and MyoD (green) in *CRY2*$^{+/+}$ and *CRY2*$^{-/-}$ primary myoblasts cultured in growth medium. The scale bar represents 100 μm. (C) Percentage of PAX7^{+}/MyoD^{+} cells. P values determined by unpaired Student's t-test. *****$P < 0.05$. n = 3 mice in each group. Mice used to start the experiment were pooled at 6 – 8 weeks of age. Data are presented as mean ±SD.

Supplemental Figure 4

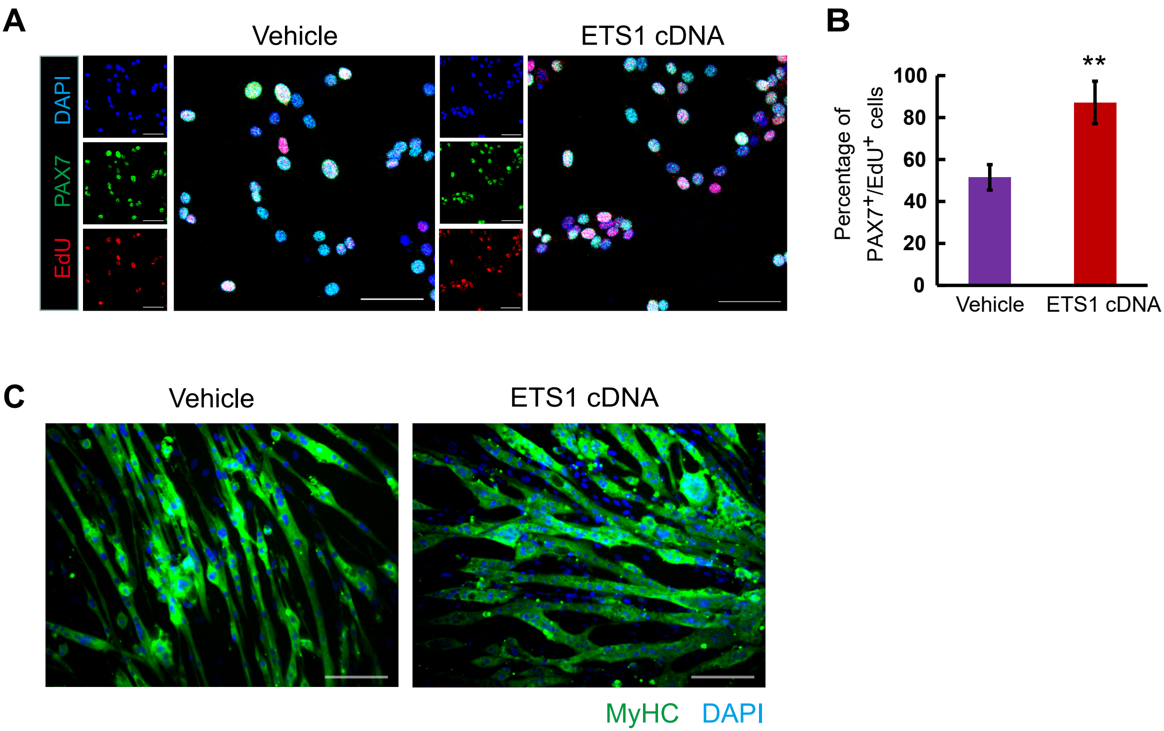

Supplemental Figure 4. ETS1 regulates satellite cells proliferation. (A) Immunostaining of PAX7 (green) and EdU (red) in wild-type C57BL/6 primary myoblasts transduced with lentivirus overexpressing ETS1 cDNA or empty vector. The scale bar represents 50 μm. (B) The percentage of $PAX7^{+}/EdU^{+}$ cells. (C) Immunostaining of MHC (green) in primary myoblasts transduced with lentivirus overexpressing ETS1 cDNA or empty vector cultured in differentiation medium for 6 days. The scale bar represents 100 μm. P values determined by unpaired Student's t-test. n = 3 in each group. Mice used to start the experiment were pooled at 6 – 8 weeks of age.**$P < 0.01$. Data are presented as mean ± SD.

Supplemental Figure 5

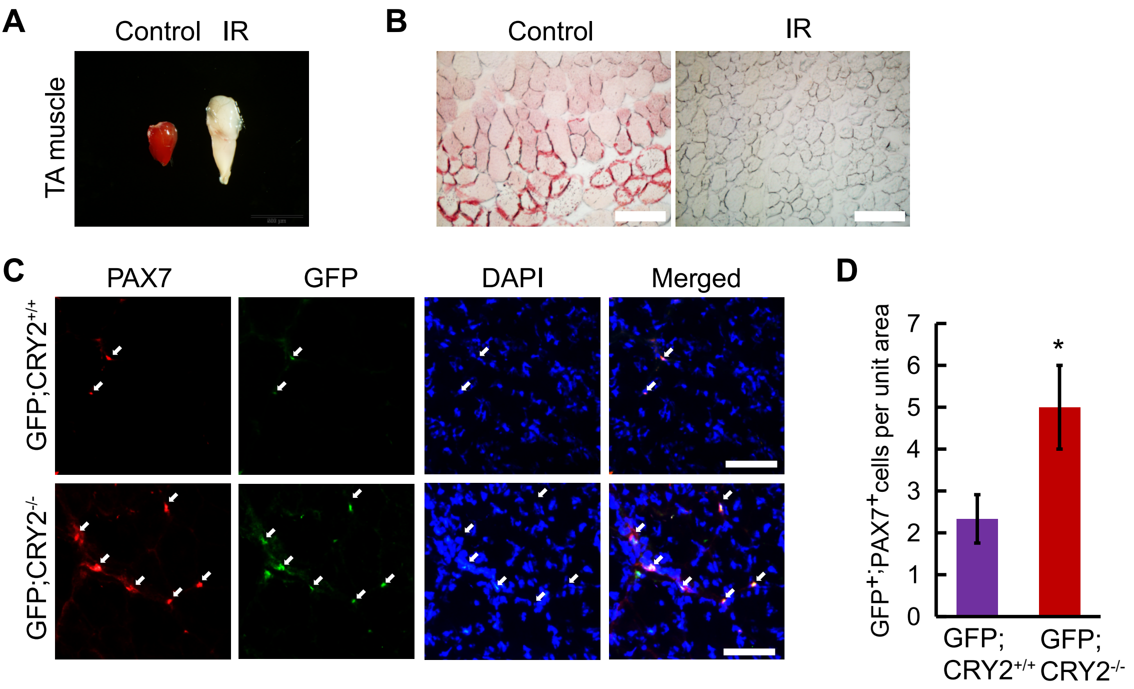

Supplemental Figure 5. Deletion of CRY2 improves engraftment and survival of satellite cells in the injured muscle. (A) Image of TTC staining for the IR-injured TA muscle and the contralateral (Ctr) control muscle. The injured muscle appears swollen and pale. Scale bars: 500 μm. (B) TTC staining of frozen sections of muscles from (A). Scale bars: 50 μm. (C) After hindlimb IR injury, 1×10^6 GFP-labeled (green) *CRY2*$^{+/+}$ or *CRY2*$^{-/-}$ primary myoblasts were injected into the TA muscle. Immunostaining of PAX7 (red) for frozen sections from the TA muscles 7 days after the injury. Scale bars: 50 μm. (D) Quantification of the number of GFP$^+$/PAX7$^+$ cells in TA muscles injected with GFP-labeled *CRY2*$^{+/+}$ or *CRY2*$^{-/-}$ primary myoblasts. P values determined by unpaired Student's t-test. n = 3 in each group. Mice used to start the experiment were pooled at 6 – 8 weeks of age.*P<0.05. Data are presented as mean ± SD.

Supplemental Figure 6

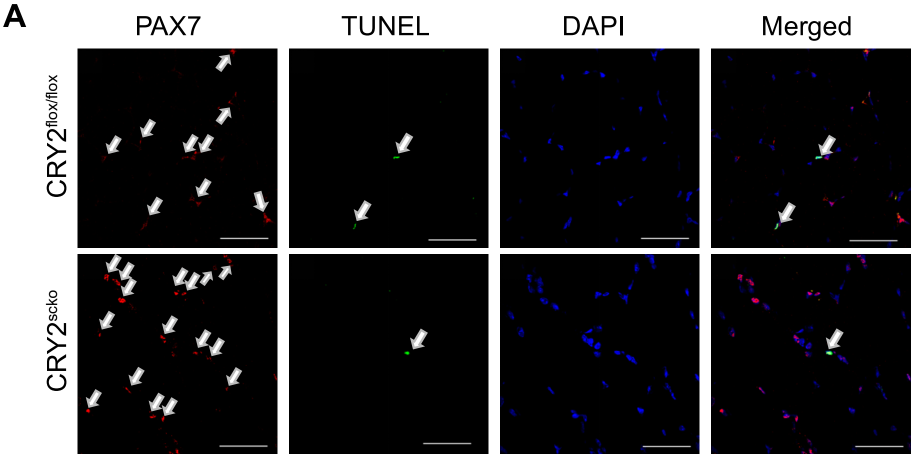

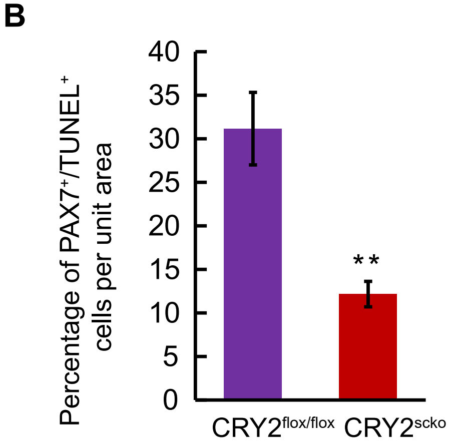

Supplemental Figure 6. Loss of CRY2 reduces apoptosis of satellite cells. (A) Representative image showing $PAX7^{+}$ and $TUNEL^{+}$ satellite cells (white arrow) in TA muscles 7 days after ischemia-reperfusion injury. PAX7 (red);TUNEL (green);DAPI (blue). Scale bar: 50 μm. (B) Percentage of $PAX7^{+}$/$TUNEL^{+}$ cells per unit area (0.04 mm^2). n = 3 in each group. Mice used to start the experiment were pooled at 6 – 8 weeks of age. P values determined by unpaired Student's t-test.**P<0.01. Data are presented as mean ± SD.

Supplemental Table 1: Sequences of qPCR primers for clock related genes.

Gene	Forward	Reverse
CRY1	GGTTGCCTGTTTCCTGACTCGT	GACAGCCACATCCAACTTCCAG
CRY2	GGACAAGCACTTGGAACGGAAG	ACAAGTCCCACAGGCGGTAGTA
CLOCK	GGCTGAAAGACGGCGAGAACTT	GTGCTTCCTTGAGACTCACTGTG
BMAL1	ACCTCGCAGAATGTCACAGGCA	CTGAACCATCGACTTCGTAGCG
PER1	GAAACCTCTGGCTGTTCCTACC	AGGCTGAAGAGGCAGTGTAGGA
PER2	CTGCTTGTTCCAGGCTGTGGAT	CTTCTTGTGGATGGCGAGCATC
PER3	CACAGACATCGAAGGAGGTGCT	CTTACACGCCACGGCAACACTT
DBP	ACACCGCTTCTCAGAGGAGGAA	TCTCGACCTCTTGGCTGCTTCA

Supplemental Table 2: Sequences of qPCR primers for myogenesis related genes.

Gene	Forward	Reverse
PAX7	CAGTGTGCCATCTACCCATGCTTA	GGTGCTTGGTTCAAATTGAGCC
MyoD	TGGGATATGGAGCTTCTATCGC	GGTGAGTCGAAACACGGATCAT
Myh3	ACATCTCTATGCCACCTTCGCTAC	GGGTCTTGGTTTCGTTGGGTAT
MHCI	CTTCTACAGGCCTGGGCTTAC	CTCCTTCTCAGACTTCCGCAG
MHCIIa	TTCCAGAAGCCTAAGGTGGTC	GCCAGCCAGTGATGTTGTAAT
MyHCIIb	CTTGTCTGACTCAAGCCTGCC	TCGCTCCTTTTCAGACTTCCG
MHCIIx	GAAGAGTGATTGATCCAAGTG	TATCTCCCAAAGTTATGAGTACA
F4/80	ACAAGTGTCTCCCTCGTGCT	AACATGTGCTTTCCACAGTC
GAPDH	CATCACTGCCACCCAGAAGACTG	ATGCCAGTGAGCTTCCCGTTCAG

Supplemental Table 3: Primers of CRY2 and ETS1.

Gene	sequence
Overexpression-CRY2-F	ACATGCATGCGCCACCATGGCGGCGGCTGCTGTGGTG
Overexpression-CRY2-R	ATAAGAATGCGGCCGCTCAGGAGTCCTTGCTTGCTGGCTCTT
Overexpression-ETS1-F	ACATGCATGCGCCACCATGAAGGCGGCCGTCGATC
Overexpression-ETS1-R	ATAAGAATGCGGCCGCCTAGTCAGCATCCGGCTTTA
Cas9-CRY2-F	CACCGCCGCCATCGCCGCCTGGACNGG
Cas9-CRY2-R	AAACGTCCAGGCGGCGATGGCGGC

Supplemental Table 4:PCR primers of PAX7 promoter .

Gene	Forward	Reverse
Primer1	AAGGGGAGCCAGTCAAAATATGC	ATCTGTCTGTCGTCTGTCCATC
Primer2	GATGGACAGACGACAGACAG	GAGGCCAAGTGGTTTTAGACTC
Primer3	GAGTCTAAAACCACTTGGCCTC	TTCTTGGAGGGGGTCAGTCT
Primer4	AGACTGACCCCCTCCAAGAA	CTCTCAGAGATCCCACAATTCTG
Primer5	AACAGAATTGTGGGATCTCTGAG	GGGAATAACCTCTCCAGCTC
Primer6	GAGCTGGAGAGGTTATTCCC	CTATCTCTGGCCTCCTGGACTA
Primer7	TAGTCCAGGAGGCCAGAGATAG	AATCTCCAACTCGACCTCG
Primer8	CGAGGTCGAGTTGGAGATT	GCGAGGGGGCGCGAGCGAT
